# Occurrence and Probabilistic Risk Assessment of Fumonisin B1, Fumonisin B2 and Deoxynivalenol in Nixtamalized Maize in Mexico City

**DOI:** 10.3390/toxins12100644

**Published:** 2020-10-06

**Authors:** Ixchel Gilbert-Sandoval, Sebastiaan Wesseling, Ivonne M. C. M. Rietjens

**Affiliations:** Division of Toxicology, Wageningen University & Research, 6708 WE Wageningen, The Netherlands; sebas.wesseling@wur.nl (S.W.); ivonne.rietjens@wur.nl (I.M.C.M.R.)

**Keywords:** fumonisins, deoxynivalenol, nixtamalized maize, exposure assessment, risk assessment, Mexico

## Abstract

Fumonisins (FB1+FB2) and deoxynivalenol (DON) are mycotoxins produced by *Fusarium* species that might be present in maize and maize products. Knowledge on their occurrence in nixtamalized maize from Mexico together with an accompanying risk assessment are scarce, while nixtamalized maize is an important food in Mexico. This study presents the occurrence of FB1 + FB2 and DON in nixtamalized maize samples collected in Mexico City and analyses their distribution and resulting estimated daily intake for Mexican consumers by a probabilistic approach using a two-dimensional Monte-Carlo simulation. The results obtained reveal that for FB1 + FB2, 47% of the Mexican men and 30% of the Mexican women might exceed the provisional tolerable daily intake (PMTDI) of 2 µg/kg bw/day for fumonisins and for DON, 9% of men and 5% of women would be exceeding the PMTDI of 1 µg/kg bw/day, corresponding to the high consumers. The results raise a flag for risk managers in Mexico, to consider regulations and interventions that lower mycotoxin levels in nixtamalized maize for human consumption.

## 1. Introduction

Infection by *Fusarium* spp. is a common contamination occurring in maize, which can result in the production of different *Fusarium* toxins that may end up as food and feed contaminants [1]. Among the *Fusarium* toxins, the B-series fumonisins are the most prevalent mycotoxins in maize and its products. The B-series of the fumonisins are modified sphingoid bases mainly consisting of fumonisin B1 (FB1), fumonisin B2 (FB2) and fumonisin B3 (FB3) [2]. FB1 is regarded as the major fumonisin because of its high occurrence, being reported to occur at more than two thirds of the sum of FB1, FB2 and FB3 [2,3].

Although evidence for adverse health effects of fumonisins in humans is limited, the main concern is their potential to contribute to cancer development through lipid metabolism disruption via the inhibition of ceramide synthase [2]. FB1 has been classified by the International Agency for Research on Cancer (IARC) as a group 2B agent, possibly carcinogenic to humans [4]. Other important concerns connected to fumonisin exposure are related to impaired growth in children and increased neural tube defects [5]. Based on liver toxicity in a short-term dose-response study in male transgenic mice with a BMDL10 (lower confidence limit of the Benchmark Dose causing 10% extra effect above background levels) of 0.165 mg/kg body weight (kg bw)/day and renal toxicity in a 90-day rat study, a provisional maximum tolerable daily intake (PMTDI) was set for FB1, FB2 and FB3, alone or in combination of 2 µg/kg bw/day by the Joint FAO/WHO committee of Food Additives (JECFA) [6].

A second important group of *Fusarium* toxins are the Type B trichothecenes, with deoxynivalenol (DON) being the one most frequently encountered in food [3]. In humans, acute exposure to DON is associated with gastrointestinal disorders [7], while reduced body weight gain is considered the critical effect in chronic exposure in experimental animals [8]. Based on a NOAEL of 0.1 mg/kg bw/day for reduced body weight gain in mice, a tolerable daily intake (TDI) and a provisional maximum TDI (PM(TDI)) of 1 µg/kg bw/day for DON and its acetylated derivatives were set by the European Food Safety Authority (EFSA) and JECFA, respectively [8,9].

In Mexico and Central American countries like Guatemala, maize is a staple food that is mainly consumed after an alkaline treatment (nixtamalization) in the form of different baked products (e.g., tortillas). Nixtamalization may reduce the content of several mycotoxins, including fumonisins and DON [5,10]. Nevertheless, mycotoxin reduction might not be enough given the high consumption of nixtamalized maize products in this area. As a result, consumption of fumonisins and DON may exceed the health-based guidance values mentioned.

Despite guidelines and standards defined internationally through the Codex General Standard for Contaminants and Toxins in Food and Feed via the Codex Alimentarius Commission established by the Food and Agriculture Organization of the United Nations (FAO) and the World Health Organization (WHO), in many of the high-maize consumption areas of the world, regulation is either lacking or not enforced [11,12]. In Mexico, mycotoxin regulation in nixtamalized maize and its products is only implemented for aflatoxins. Correspondingly, few risk assessments of mycotoxin exposure resulting from the consumption of nixtamalized maize are available, while to the best of our knowledge, risk assessments for DON exposure from consumption of nixtamalized maize are absent [13,14].

Hence, the aim of the present paper was to provide insight in the occurrence of FB1, FB2 and DON in nixtamalized maize and to perform an accompanying exposure and risk assessment. Traditionally, dietary exposure assessments have been done based on a deterministic approach by multiplying average consumption rates by average contaminant concentrations in food [15]. In order to reflect variability in the exposure assessment and consider the uncertainty associated, a probabilistic approach using a two-dimensional (second order) Monte-Carlo simulation was used. The 95th percentile EDI values were compared to the PMTDI established for the sum of FB1 and FB2 (FB1 + FB2) and the TDI established for DON.

## 2. Results

### 2.1. Occurrence of FB1, FB2 and DON in Nixtamalized Maize Samples

**Figure 1:**
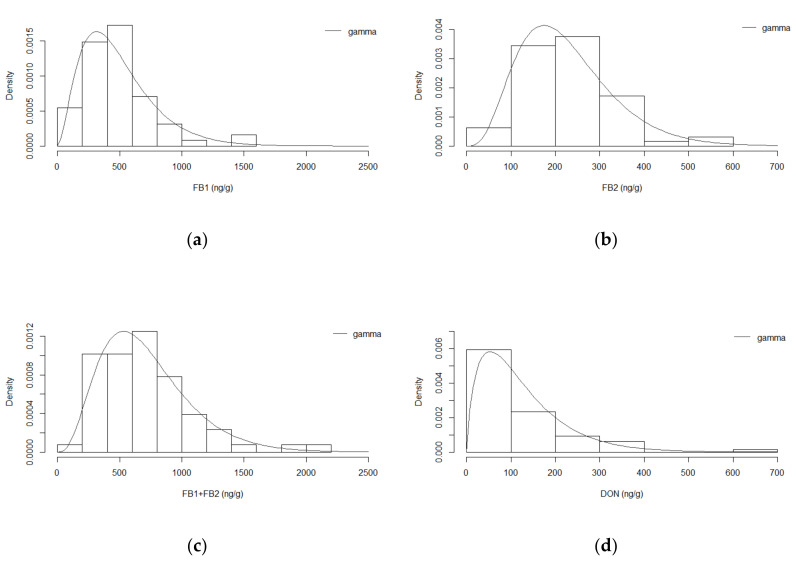
Relative frequency histogram of the mycotoxins in the nixtamalized maize samples; superimposed on the histogram is the density curve of the gamma distribution: (**a**) fumonisin B1 (FB1); (**b**) fumonisin B2 (FB2); (**c**) sum of FB1 and FB2 (FB1 + FB2); (**d**) deoxynivalenol (DON). The bars present the actual experimental data frequency densities and the line presents the fitted mathematical gamma distributions that appeared to describe the experimental data best.

Figure 1 shows the relative frequency histogram of the mycotoxin levels in the nixtamalized samples. FB1 was detected in 63 out of the 64 samples (98%) with levels ranging from 79–1589 ng/g, with a mean and median of 488 ng/g and 440 ng/g, respectively. FB2 was present in all samples with levels ranging from 24 to 524 ng/g, with a mean and median of 225 ng/g and 216 ng/g, respectively. DON was detected in 45 out of the 64 samples (70%) at levels ranging from 43 to 658 ng/g, with a mean and median of 160 and 136 ng/g, respectively. The sample with the highest concentration of FB1 (sample #54) did not correspond to the sample with the highest concentration of FB2 (sample #51) or DON (sample #49). Complete datasets are shown in the Supplementary Materials (Table S1).

### 2.2. Probabilistic Exposure Assessment

#### 2.2.1. Exposure Assessment Model

The Estimated Daily Intake (EDI) is a function of the mycotoxin concentration in the nixtamalized samples times the nixtamalized maize consumption divided by the body weight. In order to perform a probabilistic exposure assessment using a two-dimensional Monte-Carlo framework, distributions for each of these datasets were parameterized to reflect variability and uncertainty.

#### 2.2.2. Fitting Distributions to Experimental Data

The experimental data were fitted to mathematical equations that describe the distributions. In general, the goodness-of-fit plots showed that among the continuous probability distributions evaluated (lognormal, Weibull and gamma), the gamma model provided the best fit to the data, with the lowest AIC and BIC values, indicating its adequacy (Figures S1–S4 and Table S2 in the Supplementary Materials). In Figure 1, the gamma distributions thus obtained are presented and appear to fit the experimental data well.

**Figure 2:**
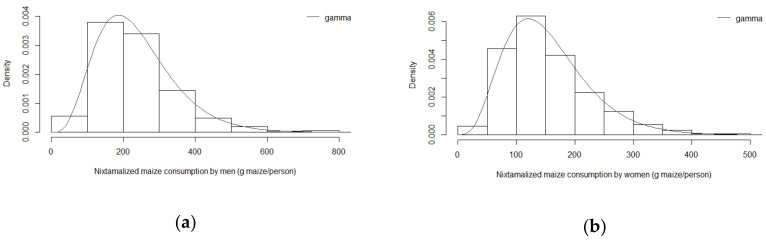
Relative frequency histogram of the total nixtamalized maize consumption by (**a**) men and (**b**) women; superimposed on the histogram is the density curve of the gamma distribution. The bars present the frequency density distributions obtained as the sum of the distributions of the consumption of individual products (tortillas, tacos, antojitos and chilaquiles) reported by Wall-Martinez [16]. The line presents the fitted mathematical gamma distributions that appeared to describe the dataset best.

To enable a probabilistic exposure assessment also distributions for actual consumption of the nixtamalized maize products are required. Figure 2 presents the sum of the reported distributions for the consumption of individual nixtamalized maize products (i.e., tortillas, tacos, antojitos and chilaquiles) by men and women, respectively. Moreover, these data were best described by the gamma distribution shown by the goodness-of-fit plots and the lowest AIC and BIC values indicating its adequacy (Figures S5 and S6 and Table S3 in the Supplementary Materials). The distributions of the body weight in men and women reported by Wall-Martinez et al. [16] were reported as gamma distributions and used as such, with the graphical representation of these distributions presented in the Supplementary Materials (Figures S7 and S8).

#### 2.2.3. Modeling Uncertainty Parameters on Each Variable of the Exposure Model

Uncertainty was simulated by a parametric bootstrap resampling of the parameters obtained from fitting distributions to the datasets (i.e., mycotoxin concentration, consumption, body weight). As described in the previous step, all datasets fitted gamma distributions, hence the shape and rate of each dataset was bootstrap resampled to derive a 95% confidence interval. Table 1 and Table 2 show the parameters obtained from fitting a gamma distribution to the datasets before and after bootstrap resampling the mycotoxin levels (Table 1), and the total nixtamalized maize consumption and body weight (Table 2).

#### 2.2.4. Integrating Uncertainty and Variability in a Two-Dimensional Monte-Carlo Simulation Framework

**Figure 3:**
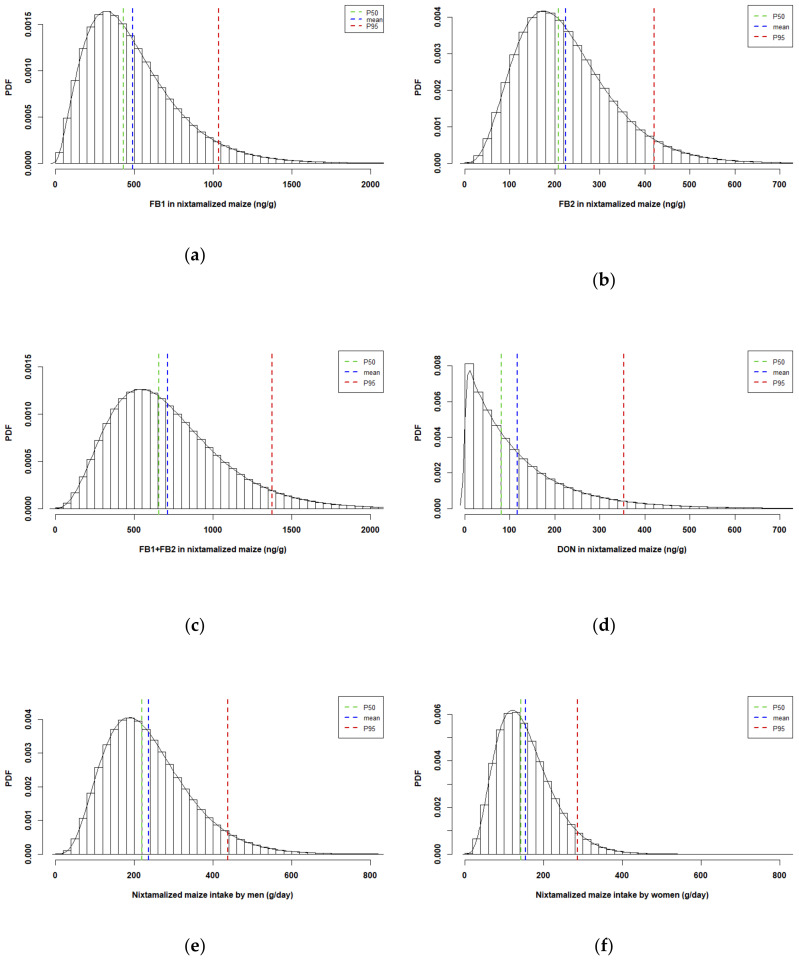
Probability density function (PDF) plots obtained after one-dimension Monte-Carlo simulation representing variability of the mycotoxins in the nixtamalized maize (ng/g): (**a**) FB1, (**b**) FB2, (**c**) sum of FB1 and FB2 (FB1 + FB2), and (**d**) DON and the nixtamalized maize consumption by (**e**) men and (**f**) women. Blue dotted lines represent the mean, green dotted lines the median (P50), and the red dotted lines the 95th percentile (P95).

In this step, the previously defined parameters of the gamma distributions for each dataset were randomly sampled with a classic Monte-Carlo simulation, resulting in the one-dimensional probability density function (PDF) plots for variability of the mycotoxin levels in the nixtamalized maize and the nixtamalized maize consumption presented in Figure 3.

Likewise, the parameters obtained by bootstrapping were also randomly sampled from a gamma distribution with a Monte-Carlo simulation. For a two-dimensional Monte-Carlo, each variability and each uncertainty parameter were randomly sampled and combined, leading to a matrix of variability and uncertainty parameters. The parameters thus obtained are presented in Table 3 for the mycotoxin concentration in the nixtamalized maize samples and in Table 4 for the total nixtamalized maize consumption and body weight. The variable inputs including uncertainty are also reflected by the integral of the PDF, which is graphically represented by the cumulative distribution function (CDF) plots in the Supplementary Materials (Figures S9 and S10). Body weight PDF and CDF plots are also displayed in the Supplementary Materials (Figure S11).

#### 2.2.5. Obtaining the EDI Values by Performing the Arithmetic Operation Defined for the Exposure Assessment Model

The final step represents the calculation of the EDI for FB1, FB2, FB1 + FB2, and DON resulting from the consumption of nixtamalized maize by men and women. The EDI values are computed from the two-dimensional Monte-Carlo simulation of the (i) mycotoxin levels in nixtamalized maize, (ii) the total nixtamalized maize consumption, and (iii) the body weight. Table 5 shows the parameters describing the distribution for the EDI values thus obtained, including both variability and uncertainty.

### 2.3. Risk Assessment

Table 5 shows that the P95 of the EDI for FB1 and FB1 + FB2 by men exceeded the PMTDI for fumonisins (2 µg/kg bw/day) to the largest extent amounting to 2- and 2.8-fold the PMTDI, respectively. In women, the P95 of the EDI for FB1 and FB1 + FB2 amounted to 1.5- and 2-fold the PMTDI, respectively. For both men and women, the EDI for FB2 was below the PMTDI for fumonisins. The EDI for DON shows that the P95 by men is 1.3-fold the (PM)TDI defined for DON of 1 µg/kg bw/day, while for women the P95 EDI is slightly lower than this health-based guidance value (0.93 µg/kg bw/day). However, the uncertainty of the P95 EDI from DON consumption by women indicates that the estimate can be above the (PM)TDI for DON of 1 µg/kg bw/day amounting to 0.70–1.20 µg/kg bw/day.

Table 5 can also be represented by CDF plots in order to obtain the probabilities of exceeding the PMTDI values for both fumonisins and DON (Figures S12 and S13 in the Supplementary Materials). In Table 6 the probabilities of exceeding the PMTDI values established for fumonisins and DON are shown.

Table 6 shows that men consuming nixtamalized maize have a 27% probability of being above the PMTDI for FB1, but only a probability of 3% of being above the PMTDI for FB2. The probability increases to 47% when fumonisins are evaluated in combination (FB1 + FB2). The EDI of DON from the consumption of nixtamalized maize by men indicates that there is a probability of 9% for being above the (PM)TDI. For women, although the scenario is similar as the one for men, the probabilities of being above the respective health-based guidance values for fumonisins and DON are lower. The estimates show that 15% of the female consumers of nixtamalized maize would have an EDI of FB1 above the health-based guidance value, while only a 0.9% would have an EDI of FB2 above the PMTDI. Considering the intake of both fumonisins (FB1 + FB2), this results in a probability of exceeding the health-based guidance value by women of 30%. In comparison, the probability of the EDI of DON from nixtamalized maize consumed by women being above the health-based guidance value is only 4%. From these data it follows that the EDI values obtained for both men and women have a higher probability of exceeding the health-based guidance values for fumonisins than for DON (Table 6).

## 3. Discussion

Nixtamalization is an important food processing method for maize in Mexico and Central America [17]. It is known to reduce the mycotoxin levels in maize and its sub-products [18]. Among these mycotoxins, fumonisins and DON are reported to be removed up to 50–99% and 72–88%, respectively [10,19]. However, the reduction of mycotoxins upon food processing does not guarantee a mycotoxin free product. The heterogenous nature and hence the initial contamination in the raw maize, as well as the nixtamalization process conditions affect the final values in nixtamalized maize based foods [18]. Moreover, because consumption patterns play an important role in the ultimate exposure, information on the natural occurrence of fumonisins and other mycotoxins, like DON, from high consumption areas is important [6,20]. In this study, we present up-to-date occurrence data on FB1, FB2 and DON from 64 nixtamalized maize flour samples obtained on the Mexican market, quantified using LC–MS/MS. The occurrence data were fitted to probabilistic models to perform a probabilistic exposure assessment, including both the variability in the mycotoxin levels and the uncertainty of the estimates obtained. The distributions for the EDI values thus obtained were used for an accompanying risk assessment.

The levels of FB1, FB2 and FB1 + FB2 detected in the samples (Table 1) were somewhat lower than the occurrence values of FB1 and FB2 reported before by De Girolamo et al. [21] who analyzed 18 nixtamalized instant maize flours, finding 17 of the samples contaminated by FB1, FB2 and FB1 + FB2 with a mean of 660, 225 and 885 ng/g, respectively. Other studies have reported higher FB1 values ranging from 210 to 1800 ng/g in nixtamalized maize dough and tortillas with a mean of 790 ng/g, and in commercial nixtamalized cornmeal a mean level of 1186 ng/g was reported [22,23]. Lower ranges have also been reported in tortilla samples with mean values of 64.2, 136.6 and 96.5 ng/g for the sum of FB1 + FB2 [13]. Hence, it was considered that the mycotoxin levels as obtained in the present study give a fair representation of the distribution of FB1, FB2 and FB1 + FB2. To the best of our knowledge, no studies on the occurrence of DON in nixtamalized maize from Mexico have been performed so far. Therefore, the data reported in this study represent the first report on the distribution of DON in nixtamalized maize samples. The distributions obtained in the present study were not normally distributed and have a highly skewed right tail, in line with what is often observed for mycotoxins and has been reported before for FB1, FB2, FB1 + FB2 and DON in products different from nixtamalized maize [6]. The effects of thermal treatment after nixtamalization (e.g., baking in a hot plate) might also affect the mycotoxin levels in the production of tortillas and the other products considered in this study (tacos, antojitos, chilaquiles) [18]; however, to present a worst case exposure and risk assessment, it was assumed that baking was not substantially affecting the mycotoxin levels.

Consumption of maize in Mexico has been long regarded as one of the highest in the world together with the maize consumption in some African and Central American countries [5,6]. Even though the consumption of nixtamalized maize (i.e., tortillas) has been reported to decrease since the 1980s in urban areas like Mexico City [24,25], the adult population that reported to consume tortillas on a daily basis is still more than 85% [26]. The 2006 estimation for the urban population still places the average consumption at 155.4g/day, contrasting with the 19.4 g/day estimated to be consumed by a person in Europe [27,28]. The study of Wall-Martinez et al. [16], although it is specific for maize consumers in the Mexican city of Veracruz, was considered adequate for providing the nixtamalized maize consumption for the assessment presented as the study gives a probabilistic consumption assessment of nixtamalized maize products for men and women.

The mean and high consumer (P95) dietary exposure estimates obtained in this study for FB1 in men and women of 1.60 (4.10) µg/kg bw/day and 1.16 (3.00) µg/kg bw/day, respectively, and for total fumonisins of 2.34 (5.64) and 1.70 (4.11) µg/kg bw/day, respectively are 2- to 5-fold higher than the international estimates for FB1 intake provided by the World Health Organization as the Global Environment Monitoring System (GEMS)/Food Cluster Diets for the cluster G05, in which Mexico is included [2]. The difference might be due to the fact that the latest GEMS/Food contaminants database (since 2011) mainly contains reports from countries with lower food-borne fumonisin concentrations (e.g., Canada, EU and Japan) [2]. In addition, the mean EDI value reported for women in urban areas of Guatemala amounts to 3.5 µg FB1/kg bw/day [29], a value that is 2-fold higher than what was estimated in the present study for women. Furthermore, estimates for total FB intake in rural communities of Guatemala resulted in EDI values for women of 0.2–5 µg total FB/kg bw/day [30]. In the Texas-Mexican border area, women were estimated to consume 0.7–9.4 µg FB1/kg bw/day [31]. The values obtained in the present study are in line with these ranges. Intake reports of DON in Mexico are scarce. The latest JECFA evaluation of DON exposure did not consider the cluster that includes Mexico as no data were submitted from countries in that cluster [9]. Two studies were found reporting DON intake estimates in Mexico for wheat and beer. The mean and P95 EDI values found in the present study for men and women of 0.39 and 1.28, and 0.28 and 0.93 µg/kg bw/day, respectively, are close to the reported EDI from wheat consumption of 0.83 µg/kg bw/day, but higher than the mean and P95 estimates from the daily intake of beer of 0.01–0.19 and 0.04–0.22 µg/kg bw/day [32,33].

The results of the EDI values in the present study reveal that more than half of the Mexican male population and one third of the female population in Mexico might be exceeding the PMTDI of 2 µg/kg bw/day for fumonisins (FB1 + FB2) already by consumption of nixtamalized maize products. Regarding the intake of DON, the probability of exceeding the (PM)TDI of 1 µg/kg bw/day is 9% and 4% for men and women, respectively. Only the consumers at the P95 are over or close to this health-based guidance value for DON. Nonetheless, there might be a health concern, as DON intake from other sources might add to the total intake and result in intake that surpasses the (PM)TDI for DON on a regular basis. The results obtained therefore corroborate the need of risk management action in Mexico to limit exposure to fumonisins and DON, including regulations on fumonisins and DON levels in nixtamalized maize, frequent monitoring and intervention practices to lower the mycotoxin levels in order to better maintain intake below the PMTDI. Consideration into the co-occurrence of other mycotoxins should not be left out. In a previous assessment of some of the samples here analyzed it was shown that 5% contained aflatoxins [14]. Altogether, the assessment reveals the need for continued risk management of mycotoxins in Mexico.

## 4. Materials and Methods

### 4.1. Occurrence of FB1, FB2 and DON in the Nixtamalized Maize Samples

#### 4.1.1. Chemicals and Reagents

Acetonitrile solutions of FB1 and FB2 mix (50 µg/mL each), DON (100 µg/mL) and U-^13^C15-DON (25 µg/mL), acetonitrile/water solutions of U-^13^C34-FB1 (25 µg/mL) and U-^13^C34-FB2 (10 µg/mL), together with the certified reference materials for FB1, FB2 and DON in maize were purchased from Romer Labs (Getzersdorf, Austria). Acetonitrile (ACN, ULC/MS grade) was purchased from Biosolve (Valkenswaard, The Netherlands), formic acid >98–100% from Emsure^®^, Merck (Darmstadt, Germany), ammonium formate >97% from Sigma-Aldrich (Zwijndrecht, The Netherlands) and anhydrous MgSO_4_ and acetic acid were bought from VWR International (Darmstadt, Germany).

#### 4.1.2. Collection of Samples

A total of 64 nixtamalized maize samples were collected from different areas in Mexico City; 22 of the samples corresponded to nixtamalized maize instant flour, and the rest was collected as dough; the samples were previously analyzed for AFB1 [14].

#### 4.1.3. Extraction

Extraction of FB1, FB2 and DON was based on the multi-targeted method based on the QuEChERs extraction described by López et al. [34] with minor modifications. Briefly, 1 g dry sample was spiked with 16 µl of ^13^C34-FB1 and 16 µl ^13^C34-FB2, and samples were then mixed with 3 mL ultra-pure water (Arium pro, Sartorius, Göttingen, Germany). After manual shaking, 4 mL of extraction solvent (ACN with 1% (*v*/*v*) acetic acid) were added. The extraction process consisted of 30 min shaking in a platform shaker (Innova 2300, New Brunswick Scientific, Nijmegen, The Netherlands). Afterwards, the sample was cleaned-up by vortexing with 1.6 g of anhydrous MgSO_4_ for 1 min, followed by centrifugation at 1960× *g* for 10 min. An aliquot of 500 µL obtained from the extraction solvent supernatant was then diluted to 1 mL with 5 µL ^13^C-DON, 45 µL ACN with 0.1% (*v*/*v*) acetic acid and 450 µL water. Samples were stored at −80°C until analysis. Before analysis, samples were vortexed, and 500 µL were filtrated using a syringeless 0.45 µm PTFE filter vials (Whatman Mini-UniPrep, GE, Buckinghamshire, United Kingdom) before LC-MS/MS analysis. Two independent extractions were performed for each sample, which were also analyzed independently.

#### 4.1.4. LC-MS/MS Analysis

The multi-targeted method based on QuEChERs extraction described by López et al. [34] was used. The sample analysis was carried out in positive electrospray mode on an LC-MS/MS consisting of a Waters Acquity UPLC coupled to a QTRAP 6500 mass spectrometer (AB SCIEX INSTRUMENTS). Chromatographic separation was obtained on a 100 × 2.1 mm, 1.8 µm particle size, UPLC HSS T3 analytical column (Waters, Milford, MA, USA). The column and sample temperature were set at 35 °C. The mobile phase used in LC-MS/MS analysis consisted of a mobile phase A containing 1 mM ammonium formate with 1% formic acid in water, and a mobile phase B consisting of 1 mM ammonium formate with 1% formic acid in methanol:water (95:3.9, *v*/*v*). The flow rate was kept at 0.4 mL/min. A gradient elution was performed as follows: 0.0 min 100% A/0% B, 2.0 min 50% A/50% B, 8.0 min 0% A/100% B, 11.0 min 100% A/0% B. Of each sample extract, 5 µl was injected. The MS analysis was conducted in MRM mode. Transitions and their parameters used for analysis of FB1, FB2 and DON are shown in Table 7.

Linearity and quantification of the mycotoxins in the samples were assessed through external calibration using isotope-labeled internal standards. Trueness was validated by using certified reference materials of the compounds in maize, containing the mycotoxins at levels of 1232 ± 152 ng/g, 282 ± 42 ng/g and 569 ± 60 ng/g for FB1, FB2 and DON, respectively. Z-scores resulting from the comparison of the certified reference values with the obtained values were 1.5, 0.5 and −1.7. The limits of detection (LOD) and limits of quantitation (LOQ) obtained for FB1, FB2 and DON using the analytical method are presented in Table 8. For recovery studies (*n* = 3), the level analyzed was 60 and 120 ng/g of FB1 and FB2, and DON, respectively. The recovery samples consisted of sample #7 spiked with the respective amounts. Recovery and relative standard deviation (RSD) were in line with the requirements specified in the EC 401/2006.

The LOQ was estimated as the lowest concentration that complied with the performance criteria/validation parameters by spiking a non-blank sample, once the LOQ was obtained the LOD was calculated using the following equation:

(1) $$LOD=\frac{3.3\times \;LOQ}{10}$$

### 4.2. Probabilistic Exposure Assessment Resulting from the Mycotoxin Levels in The Nixtamalized Maize

In order to reflect variability, a first order Monte-Carlo simulation is regularly used, while in a second order Monte-Carlo simulation, besides the variability, the uncertainty is also reflected. Because the concepts of variability and uncertainty are different, these are analyzed separately [35,36]. Following the approach by Pouillot et al. [35], the probabilistic exposure assessment consists of the following steps: (1) define the exposure assessment model, (2) fitting distributions to experimental data, (3) modeling uncertainty parameters on each variable of the exposure model, (4) integrating uncertainty and variability in a two-dimensional Monte-Carlo simulation framework and (5) obtaining the EDI values by performing the arithmetic operation defined for the exposure assessment model.

#### 4.2.1. Exposure Assessment Model 

To estimate the exposure to FB1, FB2, FB1 + FB2, and DON resulting from consuming nixtamalized maize products, the model to obtain the estimated daily intake (EDI) was as stated in the equation:

(2) $$EDI=\frac{IR\times C}{BW\;\times 1000}$$

where EDI is the daily intake of the mycotoxin expressed in µg mycotoxin/kg bw/day; IR is the daily consumption of the nixtamalized maize per person (g/person/day); C is the mycotoxin concentration in the nixtamalized maize (ng/g), and BW is the body weight of men or women (kg bw). The EDI is divided by 1000 to convert ng/kg bw to µg/kg bw. In order to reflect the variability, the IR, C, and BW are assumed to arise from probabilistic distributions based on parametric modelling of the data.

#### 4.2.2. Fitting a Distribution to the Experimental Data

The *fitdistrplus* package [37] in the R Foundation for Statistical Computing [38] was used for evaluating probability distributions that described the datasets best. To fit a parametric distribution to the datasets, these were fit to a lognormal, gamma and Weibull distribution by a maximum likelihood estimation (MLE). The adequacy of the fit was judged by the goodness-of-fit plots and by using the loglikelihood Akaike and Schwarz’s Bayesian information criteria (AIC and BIC, respectively).

##### Concentration Data

Rather than to consider the mycotoxin concentration value of each individual maize sample separately, to describe the variability, the frequency of FB1, FB2 and DON in the 64 nixtamalized maize samples were fit to probability distributions that described the data. As FB1 and FB2 are usually assessed together (FB1 + FB2), in this study the datasets of FB1 and FB2 were analyzed individually but also summed to obtain the dataset FB1 + FB2 prior to fitting to a probability distribution.

##### Nixtamalized Maize Consumption

The consumption of nixtamalized maize by Mexicans was obtained from a study on the population of Veracruz City in Mexico [16]. The study reported gamma probability distributions to describe the consumption of nixtamalized maize coming from tortillas, tacos, antojitos and chilaquiles as g of nixtamalized maize (dry basis) consumed per person by men and women (Table S4). For the present study, it was assumed that the consumption of the mentioned nixtamalized maize products was representative for the consumption by an average Mexican. This assumption was based on the consideration that Mexico City and Veracruz City are both urban areas with similar age and gender distribution [39], in addition to the fact that historical consumption of nixtamalized maize as tortillas is reported to be comparable nationwide, particularly in the center and south of the country [25,40].

It was also assumed that the distribution of each mycotoxin in the nixtamalized maize samples was the same as that in their products (tortillas, tacos, antojitos and chilaquiles). As the total consumption of nixtamalized maize per person was of interest to this study rather than the consumption per product, the probability distribution of the total nixtamalized maize consumed per person was obtained by adding up the reported gamma distributions for consumption of each product. The package coga [41] in R [38] was used to perform the summation of the gamma distributions.

##### Body Weight

Probability distributions for the body weight of Mexican men and women modelled for the population of Veracruz City in Mexico by Wall-Martinez et al. [16] were used in this study. In Table 9, the summary statistics of the body weight of men and women is shown. It was assumed that these distributions represent average Mexican men and women. The dataset was remodeled as described in the next section to display in addition to variability also uncertainty.

#### 4.2.3. Modeling Uncertainty Parameters on Each Variable of the Exposure Model

Uncertainty on each variable can be simulated by a bootstrap method [42,43]. The bootstrap is a data-based simulation method for statistical inference [43], which was used to simulate uncertainty in the parameters of the following variables: mycotoxin concentration, nixtamalized maize consumption and body weight. For this purpose, the function bootstrap resampling from the mc2d package [42] in R [38] was used with a 1000 replicates.

#### 4.2.4. Integrating Uncertainty and Variability in a Two-Dimensional Monte-Carlo Simulation Framework

The defined distributions from each dataset of the mycotoxin concentration, nixtamalized maize consumption and body weight, were randomly sampled with a classic Monte-Carlo simulation of 10,000 iterations. The uncertainty parameters obtained by the bootstrapping resampling described above were also randomly sampled with a Monte-Carlo simulation of 1000 iterations. Following the approach and the programming code developed for the two-dimensional Monte-Carlo developed by Pouillot et al. [42], for each uncertainty and variability input, 10,000 values were randomly sampled conditionally to each of the 1000 values of its uncertainty parameters. The outcome is a matrix of values reflecting variability and uncertainty for each simulated dataset.

#### 4.2.5. Obtaining the EDI Values by Performing the Arithmetic Operation Defined for the Exposure Assessment Model

Once the variability and uncertainty were integrated for each dataset by a two-dimensional Monte-Carlo simulation, the EDI was obtained by computing the arithmetic operation defined in equation 2. The variable distributions of the datasets with their uncertainty were transferred to EDI to generate the distribution for the EDI by the arithmetic operation [36,42]. The simulation of the variability and uncertainty from each dataset, their integration in a two-dimensional Monte-Carlo simulation framework, and the arithmetic operations were performed with the mc2d package [42] in R [38]. The code for all the steps is included in the Supplementary Data—Computer code repository.

### 4.3. Risk Assessment

To assess the risk, the EDI distributions obtained were compared with the PMTDI of 2 µg/kg bw/day established by JECFA for FB1 and FB2, alone or in combination [2], and the (PM)TDI of 1 µg kg bw/day for DON established by EFSA and JECFA [8,9].

## Figures and Tables

**Table 1:** Parameters and bootstrap parameters for the gamma distributions of the mycotoxin concentration in the nixtamalized maize samples (FB1, FB2, FB1 + FB2, DON).

Dataset	Parameters		Bootstrap Parameters (Median (95CI ^a^))	
	Shape	Rate	Shape	Rate
FB1	2.8	0.006	2.9 (2.1–4.0)	0.006 (0.004–0.009)
FB2	4.4	0.019	4.6 (3.4–6.5)	0.02 (0.01–0.03)
FB1 + FB2	3.9	0.006	4.0 (2.9–5.9)	0.006 (0.004–0.008)
DON	0.98	0.008	1.0 (0.6–1.5)	0.009 (0.005–0.01)

^a^ 95CI, 95% confidence interval.

**Table 2:** Parameters and bootstrap parameters for the gamma distributions of the nixtamalized maize consumption (NMC) and the body weight of men and women.

Dataset	Parameters				Bootstrap Parameters (Median (95CI ^a^))			
	Men		Women		Men		Women	
	Shape	Rate	Shape	Rate	Shape	Rate	Shape	Rate
NMC	4.8	0.02	4.6	0.030	4.8 (4.4–5.2)	0.020 (0.019–0.022)	4.7 (4.3–5.1)	0.030 (0.028–0.033)
Weight	49.9	0.68	47.7	0.72	49.9 (45.4–55.3)	0.67 (0.62–0.75)	47.8 (43.5–52.6)	0.72 (0.66–0.80)

^a^ 95CI, 95% confidence interval.

**Table 3:** Matrix showing the parameters for the distributions for mycotoxin concentrations in the nixtamalized maize samples (FB1, FB2, FB1 + FB2 and DON) including variability and uncertainty after a two-dimensional Monte-Carlo simulation.

Mycotoxin Concentration	Parameter	Mean	sd	Median	95%
FB1 (ng/g)	median	489	287	432	1036
	mean	489	289	432	1040
	2.5%	419	229	370	867
	97.5%	559	352	500	1221
FB2 (ng/g)	median	224	106	208	421
	mean	225	106	209	422
	2.5%	199	85	183	365
	97.5%	254	128	236	486
FB1 + FB2 (ng/g)	median	714	354	655	1377
	mean	714	354	656	1379
	2.5%	628	280	574	1171
	97.5%	806	433	744	1594
DON (ng/g)	median	117	118	81	353
	mean	118	119	81	354
	2.5%	90	85	56	265
	97.5%	149	159	108	464

**Table 4:** Matrix showing the parameters for the distributions of nixtamalized maize (NM) consumption by men and women and the body weight of men and women, including variability and uncertainty after a two-dimensional Monte-Carlo simulation.

Data			Men				Women		
	Parameter	Mean	sd	Median	P95	Mean	sd	Median	P95
NM consumption (g/day)	median	237	108	220	438	154	71	143	286
	mean	237	108	220	438	154	71	143	286
	2.5%	230	102	213	422	149	68	138	275
	97.5%	244	114	228	455	158	75	147	297
Body weight (kg)	median	74	10	73	92	66	10	66	83
	mean	74	10	73	92	66	10	66	83
	2.5%	73	10	72	90	65	9	65	81
	97.5%	75	11	74	93	67	10	66	84

**Table 5:** Matrix showing the parameters for the distribution of the EDI values of mycotoxins upon consumption of nixtamalized maize for men and women (µg/kg bw/day) displaying variability and uncertainty obtained after the mathematical combination of two-dimensional Monte-Carlo simulations of each dataset included in the exposure model (Table 3 and Table 4): the mycotoxin concentration, the sum of the nixtamalized maize (NM) consumption by men and women and the body weight by men and women. In bold the estimates that exceed the PMTDI ^a^.

EDI (µg/kg bw/day)			Men				Women		
	Parameter	Mean	sd	Median	P95	Mean	sd	Median	P95
**FB1**	median	1.60	1.30	1.24	**4.10**	1.16	0.95	0.90	**3.00**
	mean	1.60	1.31	1.25	**4.12**	1.16	0.95	0.90	**3.00**
	2.5%	1.38	1.07	1.07	**3.47**	0.99	0.78	0.77	**2.51**
	97.5%	1.83	1.55	1.45	**4.76**	1.33	1.14	1.05	**3.51**
**FB2**	median	0.74	0.52	0.60	1.74	0.53	0.38	0.44	1.27
	mean	0.74	0.53	0.60	1.75	0.54	0.38	0.44	1.27
	2.5%	0.65	0.45	0.53	1.53	0.47	0.33	0.38	1.11
	97.5%	0.84	0.61	0.69	1.99	0.60	0.45	0.50	1.45
**FB1 + FB2**	median	**2.34**	1.72	1.90	**5.64**	1.70	1.26	1.37	**4.11**
	mean	**2.34**	1.72	1.90	**5.64**	1.70	1.26	1.37	**4.11**
	2.5%	**2.05**	1.44	1.65	**4.86**	1.49	1.06	1.20	**3.53**
	97.5%	**2.65**	**2.03**	**2.16**	**6.45**	1.92	1.48	1.56	**4.71**
**DON**	median	0.39	0.47	0.23	**1.28**	0.28	0.34	0.17	0.93
	mean	0.39	0.47	0.23	**1.28**	0.28	0.34	0.17	0.93
	2.5%	0.29	0.34	0.16	0.96	0.21	0.25	0.12	0.70
	97.5%	0.49	0.62	0.31	**1.63**	0.36	0.46	0.22	**1.20**

^a^ PMTDI, Provisional Maximum Tolerable Daily Intake; according to JECFA fumonisins alone or in combination have a PMTDI of 2 µg/kg bw/day and DON of 1 µg/kg bw/day.

**Table 6:** % of EDI values that exceed the Provisional Maximum Tolerable Daily Intake (PMTDI) for fumonisin B1 (FB1), fumonisin B2 (FB2), the sum of FB1 and FB2 (FB1 + FB2), and deoxynivalenol (DON) in men and women.

Mycotoxin	% EDI exceeding the PMTDI ^1^ (Median [95 CI%] ^2^)	
	Men	Women
FB1 FB2 FB1 + FB2 DON	26.9 [21.0–33.6] 3.0 [1.9–5.2] 47.0 [40.0–54.4] 8.6 [5.1–14.2]	14.6 [10.5–20.1] 0.8 [0.4–1.7] 29.7 [24.5–35.3] 4.3 [2.2–8.2]

^1^ PMTDI, Provisional Maximum Tolerable Daily Intake; according to JECFA fumonisins alone or in combination have a PMTDI of 2 µg/kg bw/day and DON of 1 µg/kg bw/day. ^2^ M, median; 95% credible interval

**Table 7:** MS/MS transition settings for FB1, FB2 and DON.

Analyte	Precursor Ion (*m*/*z*)	Declustering Potential (V)	Potential Ion (*m*/*z*)	Collision Energy (V)	Cell Exit Potential (V)
FB1	722.5	40	334.4/352.3	57/55	4/12
FB2	706.4	40	336.3/318.5	53/51	8/2
DON	297.1	30	249.0/231.0	15/17	15/15

**Table 8:** Method performance parameters.

**Analyte**	**LOQ (ng/g)**	**LOD (ng/g)**	**Linearity (ng/g)**	**Recovery (%) ± RSDr ^1^ (*n* = 3)**
FB1	60	20	100–800	118 ± 4.1
FB2	60	20	100–800	110 ± 0.2
DON	120	40	200-1600	75 ± 3.8

^1^ RSDr = relative standard deviation—repeatability.

**Table 9:** Summary statistics for the kilogram body weight (kg bw) of men and women reported by Wall-Martinez et al., [16].

Group	Average (kg bw)	SD	Distribution	Shape ^1^	Scale ^1^
Men	73.3	10.31	gamma	50.64	1.44
Women	65.8	9.6	gamma	46.97	1.40

^1^ Shape and scale calculated from the average and SD reported by Wall-Martinez et al. [16].

## Supplementary Materials

The following are available online at https://www.mdpi.com/2072-6651/12/10/644/s1. Table S1: Concentrations of mycotoxins FB1, FB2 and DON in the nixtamalized maize samples analyzed. Figure S1: Goodness-of-fit plots for the distribution of the FB1 concentration in the nixtamalized maize samples. Figure S2: Goodness-of-fit plots for the distribution of the FB2 concentration in the nixtamalized maize samples. Figure S3: Goodness-of-fit plots for the distribution of the FB1 + FB2 concentration in the nixtamalized maize samples. Figure S4: Goodness-of-fit plots for the distribution of the DON concentration in the nixtamalized maize samples. Table S2: Model selection criteria based on the loglikelihood Akaike and Schwarz’s Bayesian information criteria for the mycotoxin dataset. Figure S5: Goodness-of-fit plots for the distribution of the nixtamalized maize consumed daily by men. Figure S6: Goodness-of-fit plots for the distribution of the nixtamalized maize consumed daily by women. Table S3: Model selection criteria based on the loglikelihood Akaike and Schwarz’s Bayesian information criteria for the mycotoxin dataset. Figure S7: Goodness-of-fit plots for the distribution of the body weight in men. Figure S8: Goodness-of-fit plots for the distribution of the body weight in women. Figure S9: Cumulative distribution function (CDF) plots representing the mycotoxin concentration in the nixtamalized maize. Figure S10: Cumulative distribution function (CDF) plots representing the nixtamalized maize consumption as g per day. Figure S11: Probability density function (PDF) plots representing the body weight. Figure S12: Cumulative distribution function (CDF) plots representing the mycotoxin Estimated Daily Intake (EDI) by men. Figure S13: Cumulative probability plots representing the mycotoxin Estimated Daily Intake (EDI) by women. Table S4: Summary statistics for the consumption of maize by men and women reported by Wall-Martinez et al., [16]. Supplementary Data—Computer code repository: https://doi.org/10.5281/zenodo.4031516.
