# Supplementary material for: Occurrence and Probabilistic Risk Assessment of Fumonisin B1, Fumonisin B2 and Deoxynivalenol in Nixtamalized Maize in Mexico City
Source: Toxins (Basel). 2020 Oct 6;12(10):644. doi: 10.3390/toxins12100644 (PMC7600745; doi:10.3390/toxins12100644)
Supplement: Supplementary file 1 [file toxins-12-00644-s001.pdf]

Supplementary Materials: Occurrence and Probabilistic Risk Assessment of Fumonisin B1, Fumonisin B2 and Deoxynivalenol in Nixtamalized Maize in Mexico City

Ixchel Gilbert-Sandoval, Sebastiaan Wesseling and Ivonne M.C.M. Rietjens

Table 1. Concentrations of mycotoxins fumonisin B1 (FB1), fumonisin B2 (FB2) and deoxynivalenol (DON) in the nixtamalized maize samples analyzed (mean of duplicates).

Sample	FB1 (ng/g)	FB2 (ng/g)	DON (ng/g)	Sample	FB1 (ng/g)	FB2 (ng/g)	DON (ng/g)	Sample	FB1 (ng/g)	FB2 (ng/g)	DON (ng/g)
1	357	292	<LOD	11	376	181	158	21	440	250	73
2	316	236	<LOD	12	222	141	63	22	173	125	<LOD
3	691	352	154	13	515	242	100	23	500	340	78
4	254	123	<LOD	14	382	216	81	24	543	301	<LOD
5	194	132	<LOD	15	667	310	320	25	114	117	<LOD
6	318	233	<LOD	16	267	183	43	26	130	90	<LOD
7	<LOD	24	43	17	402	167	92	27	415	209	<LOD
8	79	49	111	18	459	210	376	28	573	260	250
9	415	246	<LOD	19	979	397	355	29	482	255	92
10	669	309	85	20	439	307	55	30	168	155	<LOD
**Sample**	**FB1 (ng/g)**	**FB2 (ng/g)**	**DON (ng/g)**	**Sample**	**FB1 (ng/g)**	**FB2 (ng/g)**	**DON (ng/g)**	**Sample**	**FB1 (ng/g)**	**FB2 (ng/g)**	**DON (ng/g)**
31	217	146	<LOD	43	463	215	383	55	486	214	41
32	583	339	277	44	786	267	239	56	420	133	<LOD
33	352	298	143	45	551	140	<LOD	57	598	206	81
34	289	115	170	46	358	152	161	58	267	98	137
35	214	216	<LOD	47	606	151	57	59	994	300	182
36	218	130	62	48	642	160	120	60	942	330	74
37	398	203	75	49	1438	507	658	61	353	177	136
38	529	224	118	50	452	147	209	62	298	124	90
39	455	284	160	51	1044	524	291	63	876	262	<LOD
40	538	321	200	52	613	245	78	64	437	122	<LOD
41	691	328	143	53	244	101	154				
42	748 [a]	282	231	54	1589	482	<LOD				

[a] Sample analyzed one time for FB1. LOD, limit of detection

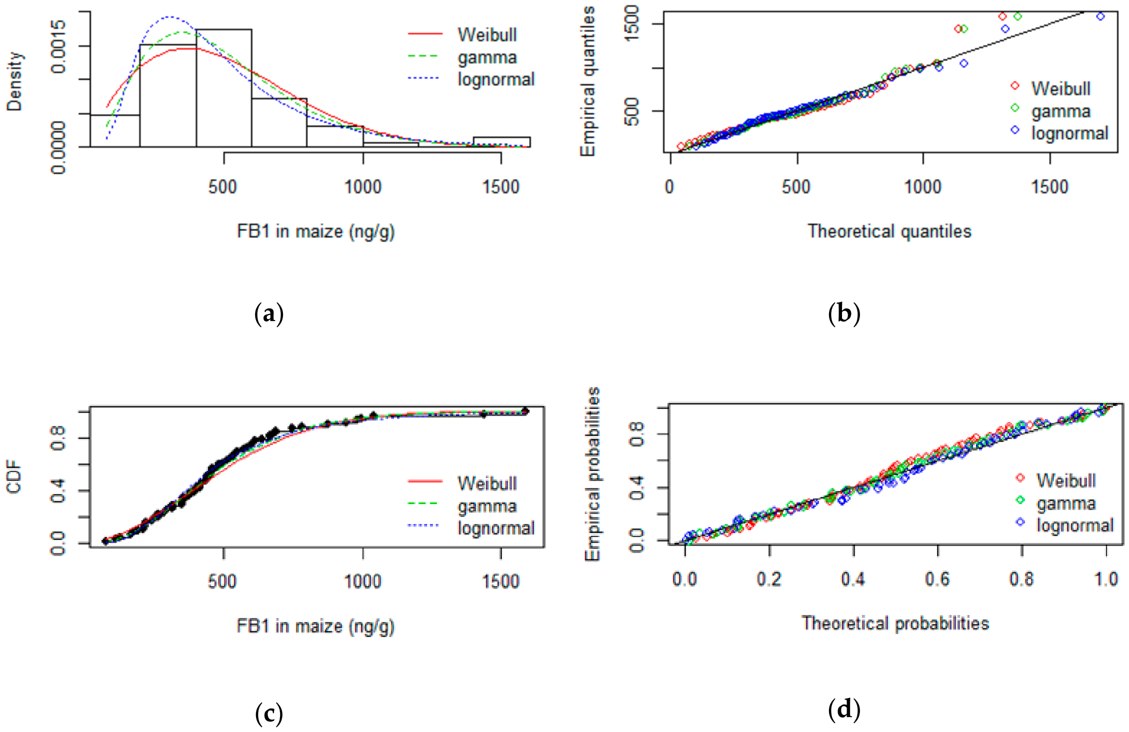

Figure S1. Goodness-of-fit plots for the distributions of the FB1 concentration in the nixtamalized maize samples. In order: (**a**) histogram of the empirical distribution data superimposed with the density function of the theoretical distribution; (**b**) plot of the quantiles of the theoretical fitted distribution (x-axis) against the empirical quantiles of the data (y-axis) (Q-Q plot); (**c**) plot of the cumulative density function of the fitted distribution (x-axis) against the empirical cumulative density function (y-axis) (P-P plot); (**d**) empirical density function of the data superimposed with the cumulative density function of the theoretical fitted distribution.

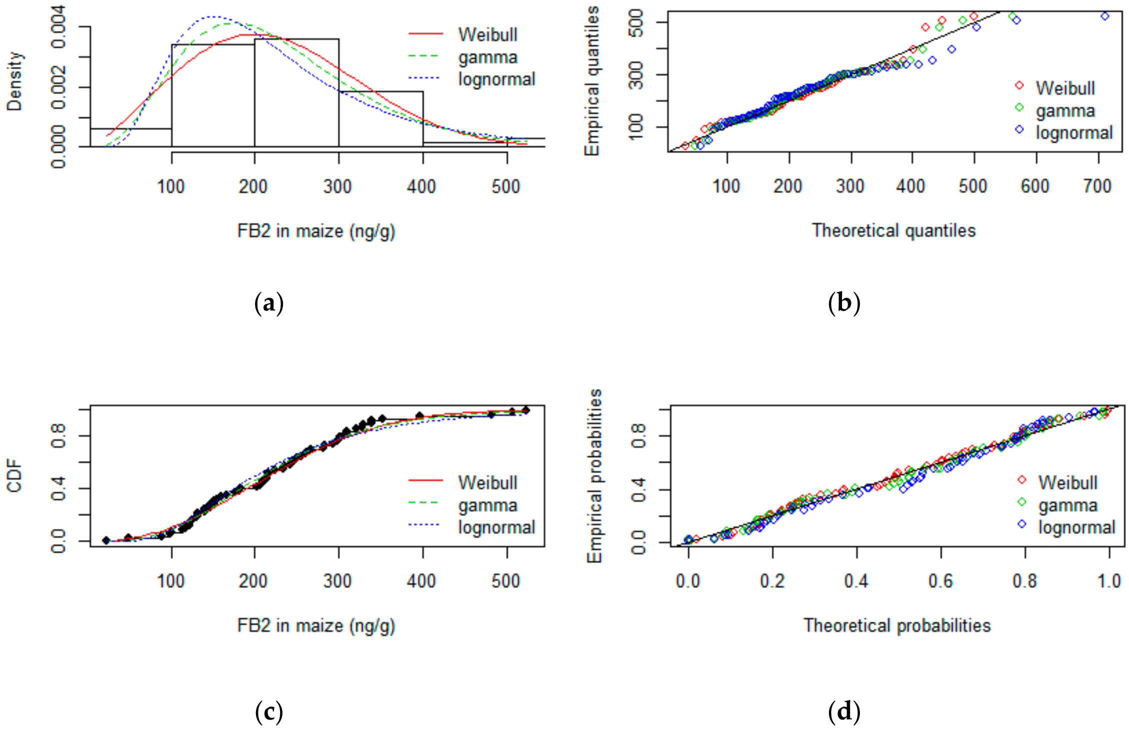

Figure S2. Goodness-of-fit plots for the distributions of the FB2 concentration in the nixtamalized maize samples. In order: (**a**) histogram of the empirical distribution data superimposed with the density function of the theoretical distribution; (**b**) plot of the quantiles of the theoretical fitted distribution (x-axis) against the empirical quantiles of the data (y-axis) (Q-Q plot); (**c**) plot of the cumulative density function of the fitted distribution (x-axis) against the empirical cumulative density function (y-axis) (P-P plot); (**d**) empirical density function of the data superimposed with the cumulative density function of the theoretical fitted distribution.

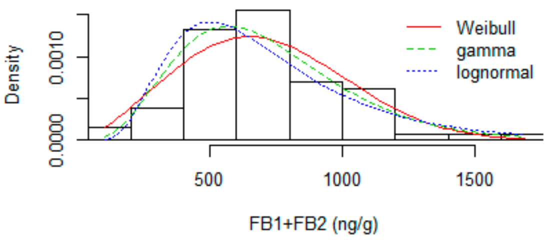

(**a**)

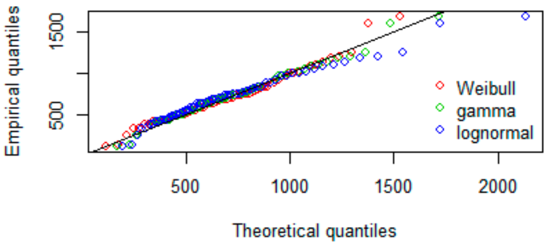

(**b**)

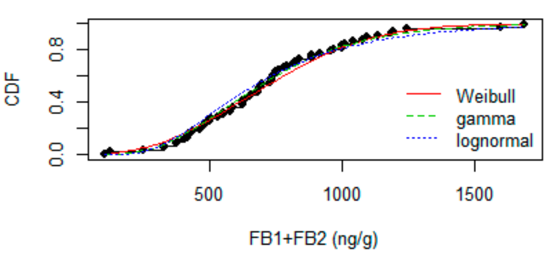

(**c**)

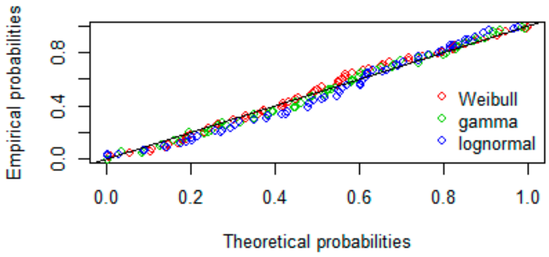

(**d**)

Figure S3. Goodness-of-fit plots for the distributions of the sum of FB1+FB2 concentration in the nixtamalized maize samples. In order: (**a**) histogram of the empirical distribution data superimposed with the density function of the theoretical distribution; (**b**) plot of the quantiles of the theoretical fitted distribution (x-axis) against the empirical quantiles of the data (y-axis) (Q-Q plot); (**c**) plot of the cumulative density function of the fitted distribution (x-axis) against the empirical cumulative density function (y-axis) (P-P plot); (**d**) empirical density function of the data superimposed with the cumulative density function of the theoretical fitted distribution.

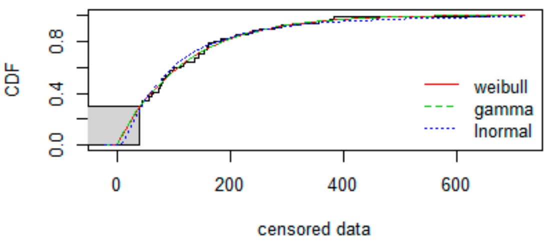

(**a**)

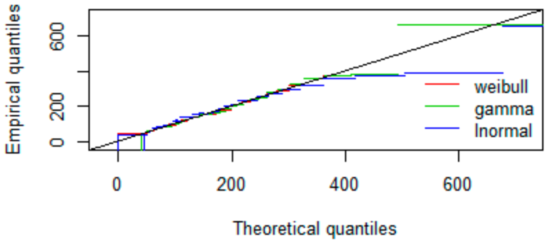

(**b**)

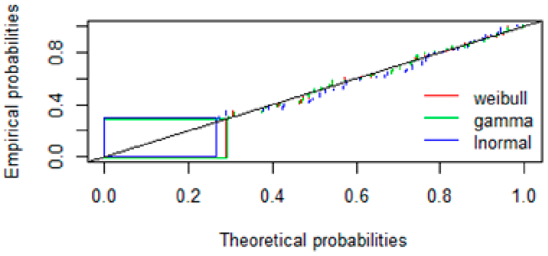

(**c**)

Figure S4. Goodness-of-fit plots for the distributions of the DON concentration in the nixtamalized maize samples. In order: (i) empirical density function of the data superimposed with the cumulative density function of the theoretical fitted distribution; (ii) plot of the quantiles of the theoretical fitted distribution (x-axis) against the empirical quantiles of the data (y-axis) (Q-Q plot); (iii) plot of the cumulative density function of the fitted distribution (x-axis) against the empirical cumulative density function (y-axis) (P-P plot).

Table S2. Model selection criteria based on the loglikelihood Akaike and Schwarz's Bayesian information criteria for the mycotoxin dataset.

Dataset	Distribution	AIC/BIC
FB1	Gamma	895.5/899.8
	Lognormal	905.5/909.8
	Weibull	896.7/901.0
FB2	Gamma	773.2/777.5
	Lognormal	781.0/785.3
	Weibull	772.9/777.2
FB1+FB2	Gamma	927.2/931.5
	Lognormal	927.5/931.8
	Weibull	932.5/936.8
DON	Gamma	602.7/607.0
	Lognormal	606.2/610.5
	Weibull	602.7/607.0

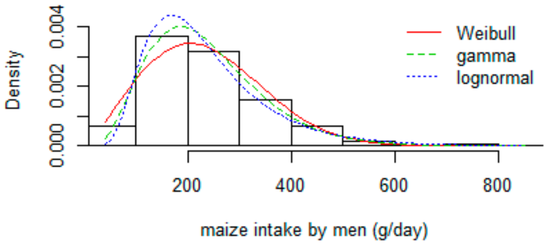

(**a**)

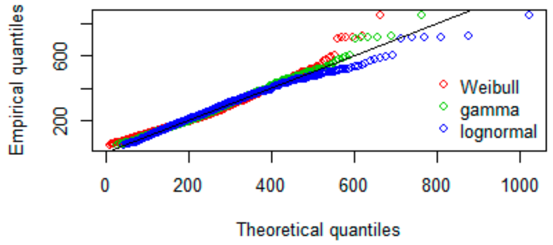

(**b**)

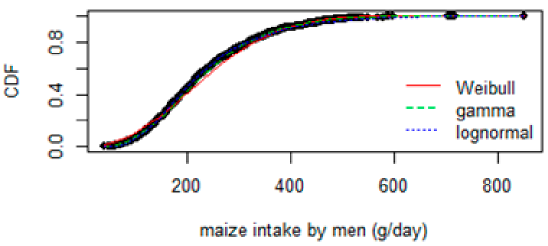

(**c**)

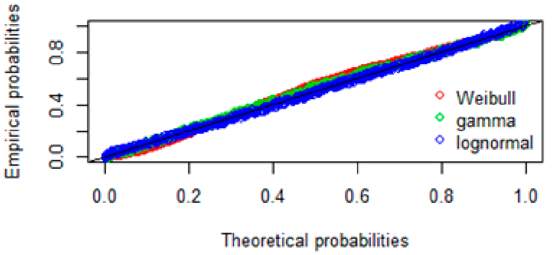

(**d**)

Figure S5. Goodness-of-fit plots for the distribution of the nixtamalized maize consumed daily by men. In order: (**a**) histogram of the empirical distribution data superimposed with the density function of the theoretical distribution; (**b**) plot of the quantiles of the theoretical fitted distribution (x-axis) against the empirical quantiles of the data (y-axis) (Q-Q plot); (**c**) plot of the cumulative density function of the fitted distribution (x-axis) against the empirical cumulative density function (y-axis) (P-P plot); (**d**) empirical density function of the data superimposed with the cumulative density function of the theoretical fitted distribution.

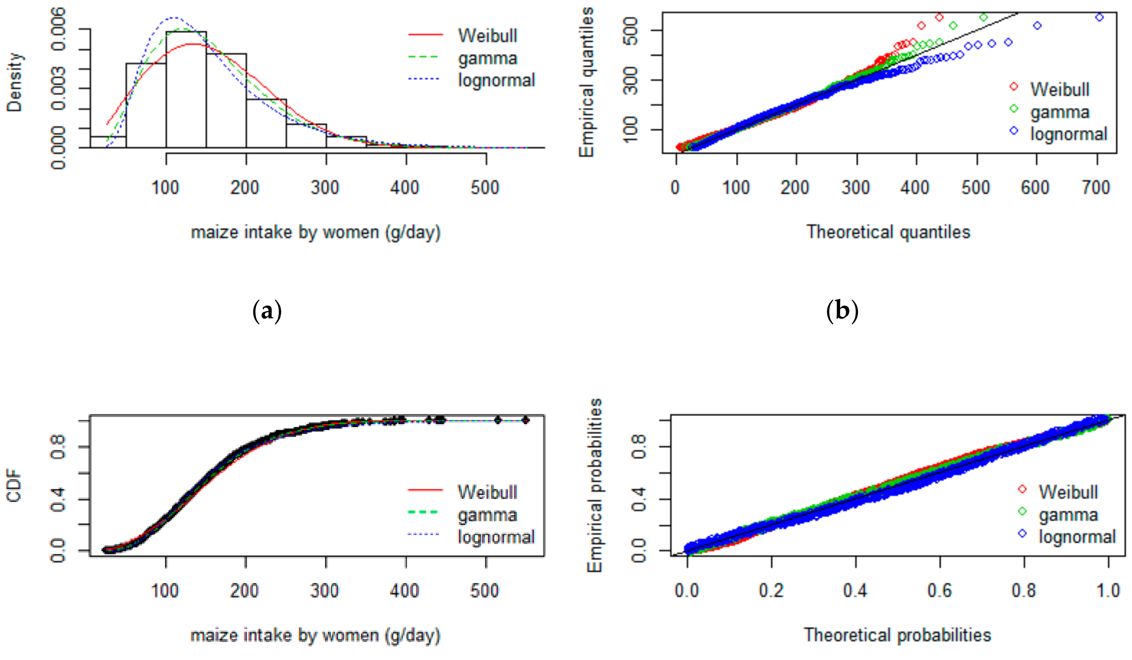

(**a**) (**b**)

(**c**) (**d**)

Figure S6. Goodness-of-fit plots for the distribution of the nixtamalized maize consumed daily by women. In order: (**a**) histogram of the empirical distribution data superimposed with the density function of the theoretical distribution; (**b**) plot of the quantiles of the theoretical fitted distribution (x-axis) against the empirical quantiles of the data (y-axis) (Q-Q plot); (**c**) plot of the cumulative density function of the fitted distribution (x-axis) against the empirical cumulative density function (y-axis) (P-P plot); (**d**) empirical density function of the data superimposed with the cumulative density function of the theoretical fitted distribution.

Table S3. Model selection criteria based on the loglikelihood Akaike and Schwarz's Bayesian information criteria for the sum of the nixtamalized maize products dataset.

Dataset	Distribution	AIC/BIC
Men	Gamma	12064.8/12074.6
	Lognormal	12051.6/12061.4
	Weibull	12169.0/12178.8
Women	Gamma	11226.0/11235.8
	Lognormal	11222.2/11232.1
	Weibull	11309.0/11318.8

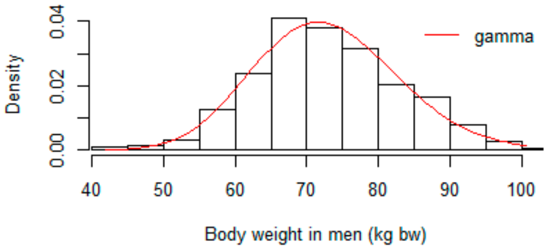

(**a**)

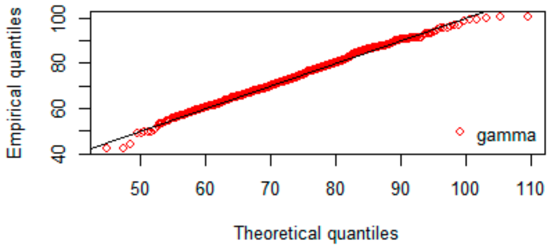

(**b**)

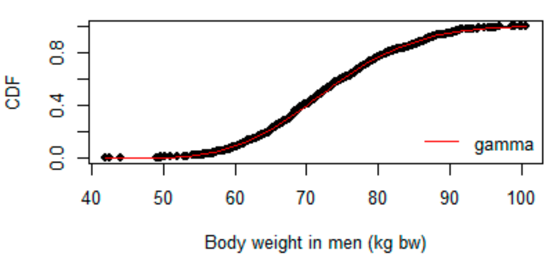

(**c**)

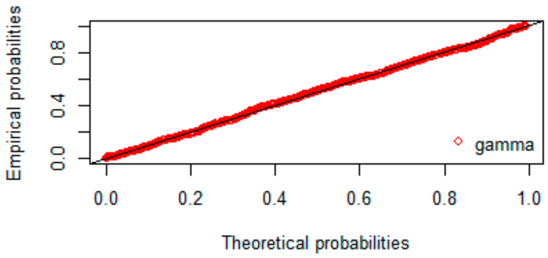

(**d**)

Figure S7. Goodness-of-fit plots for the distributions of the body weight in men. In order: (**a**) histogram of the empirical distribution data superimposed with the density function of the theoretical distribution; (**b**) plot of the quantiles of the theoretical fitted distribution (x-axis) against the empirical quantiles of the data (y-axis) (Q-Q plot); (**c**) plot of the cumulative density function of the fitted distribution (x-axis) against the empirical cumulative density function (y-axis) (P-P plot); (**d**) empirical density function of the data superimposed with the cumulative density function of the theoretical fitted distribution.

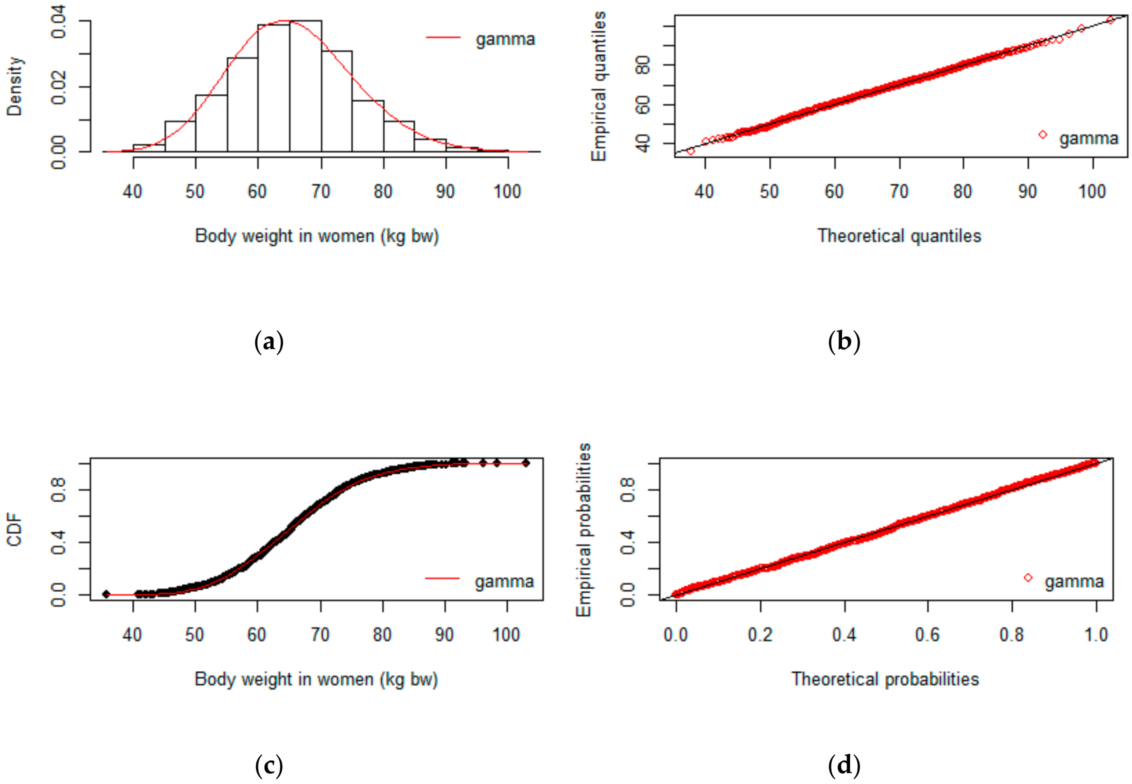

Figure S8. Goodness-of-fit plots for the distributions of the body weight in women. In order: (**a**) histogram of the empirical distribution data superimposed with the density function of the theoretical distribution; (**b**) plot of the quantiles of the theoretical fitted distribution (x-axis) against the empirical quantiles of the data (y-axis) (Q-Q plot); (**c**) plot of the cumulative density function of the fitted distribution (x-axis) against the empirical cumulative density function (y-axis) (P-P plot); (**d**) empirical density function of the data superimposed with the cumulative density function of the theoretical fitted distribution.

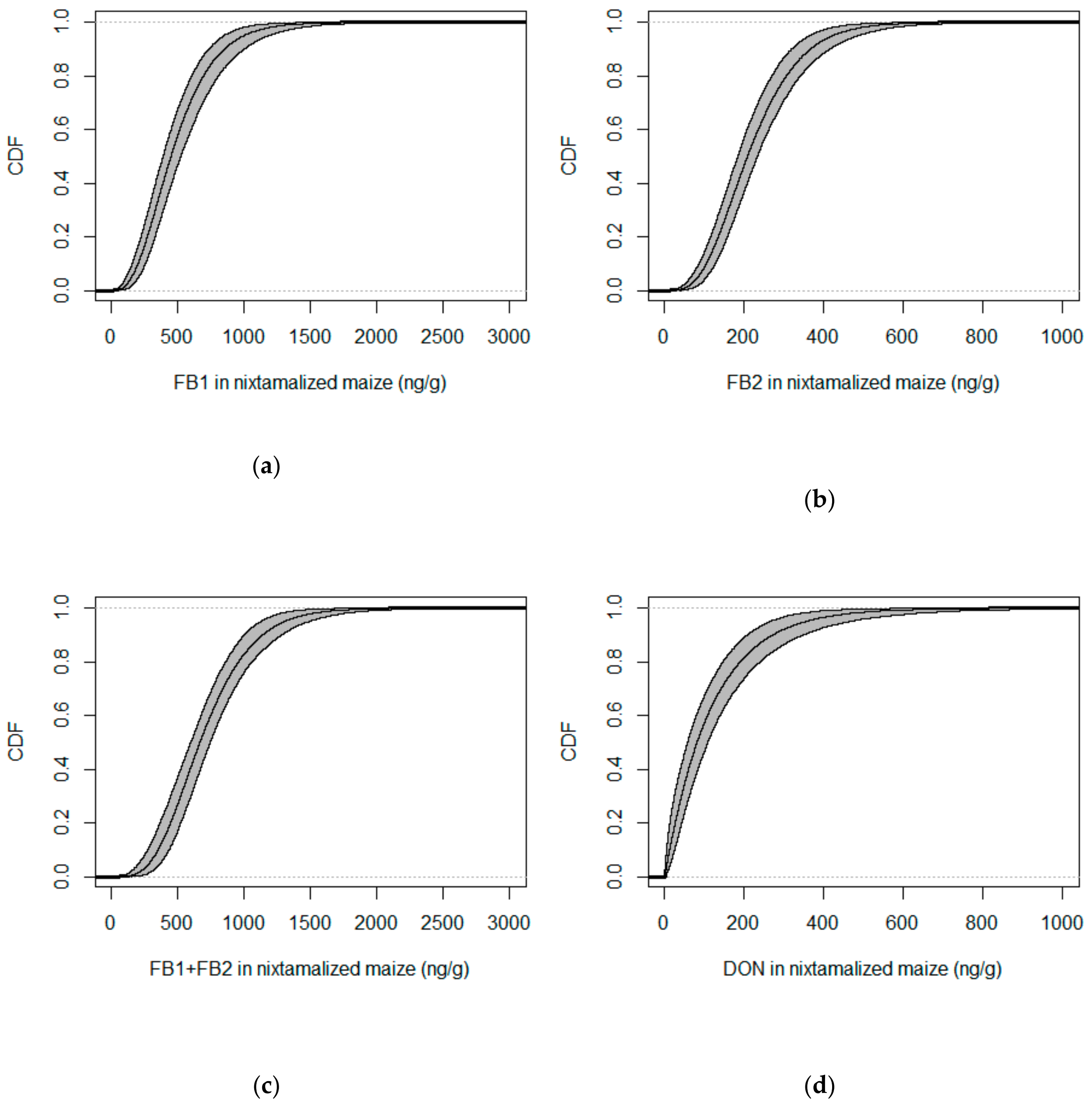

Figure S9. Cumulative distribution function (CDF) plots representing the mycotoxin concentration in the nixtamalized maize (**a**) FB1; (**b**) FB2; (**c**) FB1 + FB2; (**d**) DON. Uncertainty is indicated as the light gray band corresponding to a 95% uncertainty range.

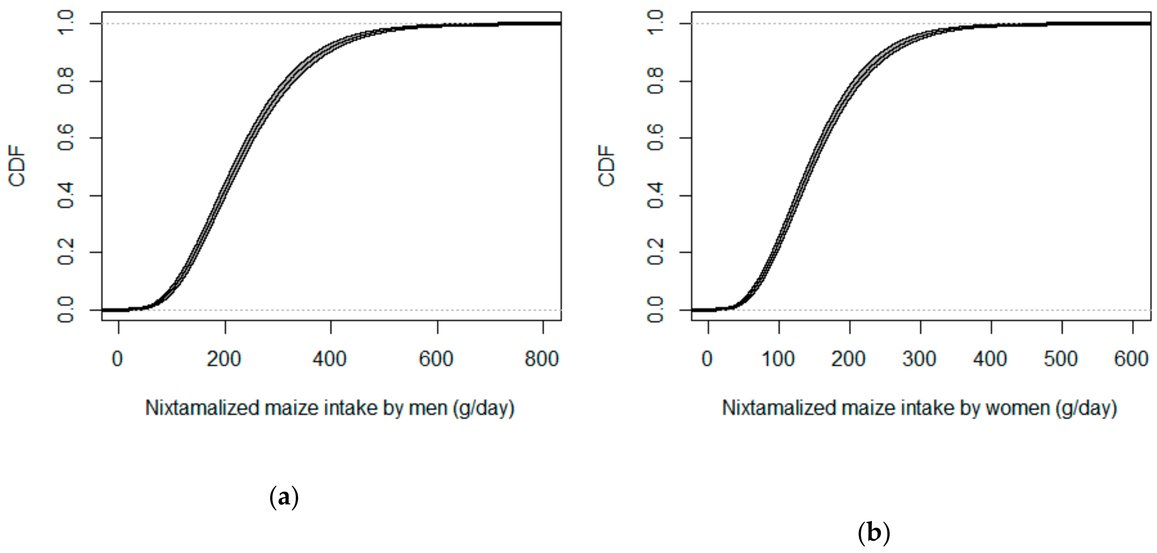

Figure S10. Cumulative distribution function (CDF) plots representing the nixtamalized maize intake as g per day, by (**a**) men and (**b**) women from the sum of nixtamalized maize products. Uncertainty is indicated as the light gray band corresponding to a 95% uncertainty range.

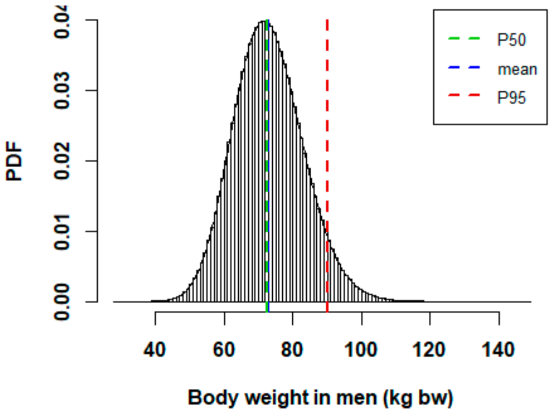

(**a**)

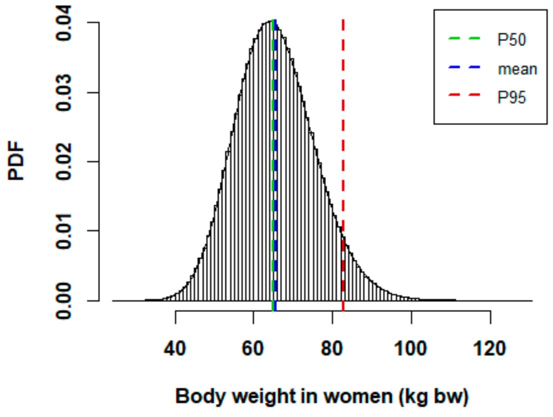

(**b**)

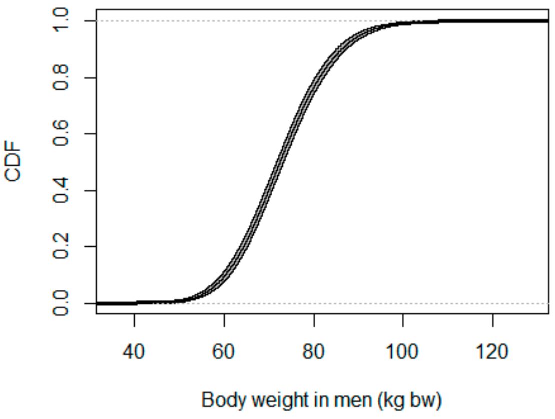

(**c**)

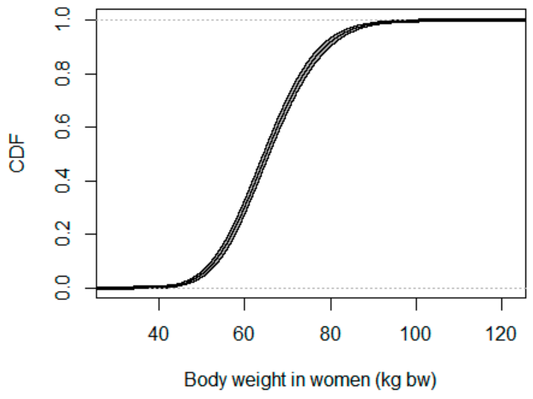

(**d**)

Figure S11. Probability density function (PDF) plots representing the body weight in: (**a**) men and (**b**) women; and cumulative distribution function (CDF) plots representing the body weight in: (**c**) men and (**d**) women; Uncertainty is indicated as the light gray band corresponding to a 95% uncertainty range.

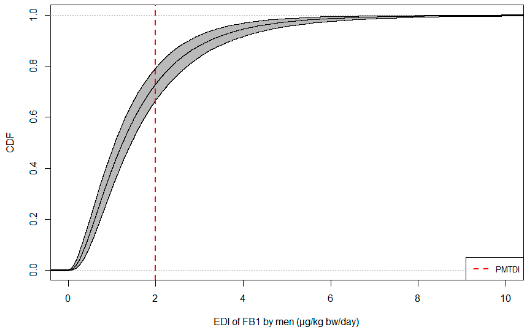

(**a**)

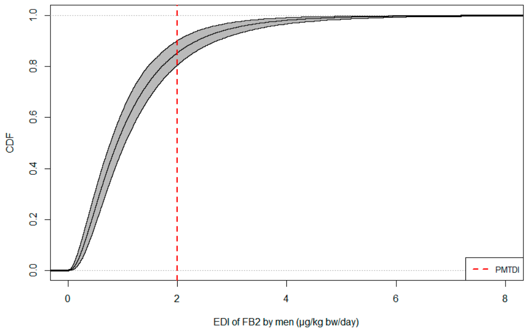

(**b**)

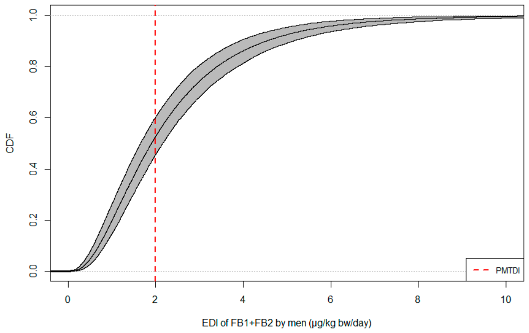

(**c**)

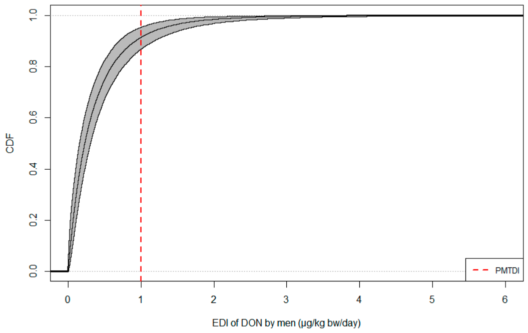

(**d**)

Figure S12. Cumulative distribution function (CDF) plots representing the mycotoxin Estimated Daily Intake (EDI) by men of (**a**) FB1; (**b**) FB2; (**c**) FB1+FB2; (**d**) DON from nixtamalized maize consumption. Uncertainty is indicated as the light gray band corresponding to a 95% uncertainty range. The Provisional Maximum Tolerable Daily Intake (PMTDI) is indicated as a red dotted line; according to JECFA fumonisins alone or in combination have a PMTDI of 2 µg/kg bw/day and DON of 1 µg/kg bw/day.

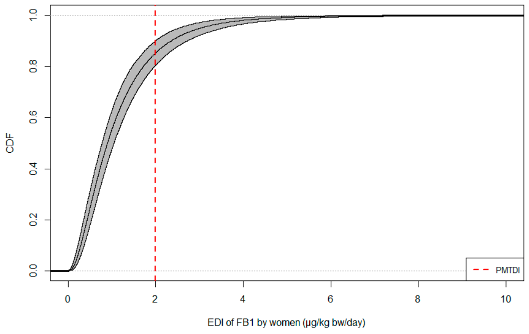

(**a**)

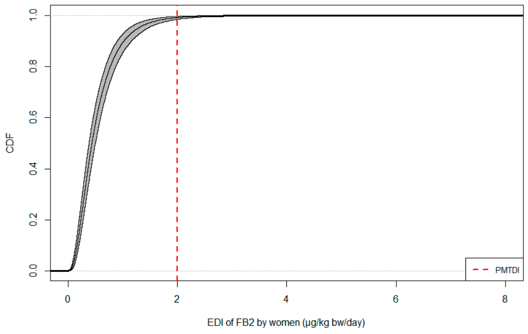

(**b**)

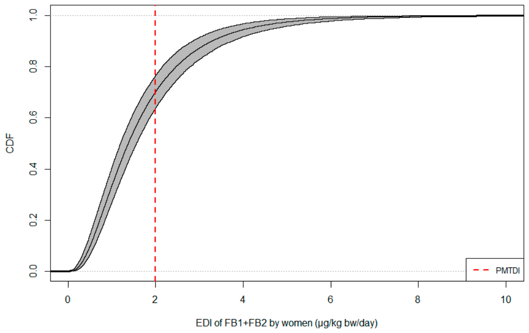

(**c**)

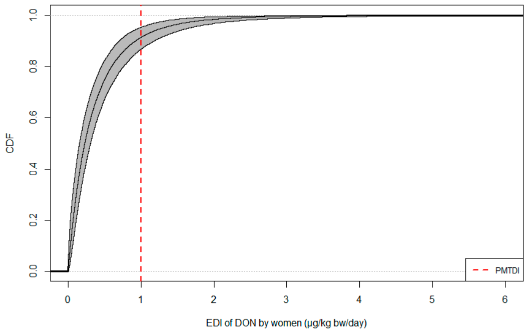

(**d**)

Figure S13. Cumulative probability plots representing the mycotoxin Estimated Daily Intake (EDI) by women of (**a**) FB1; (**b**) FB2; (**c**) FB1 + FB2; (**d**) DON from nixtamalized maize consumption. Uncertainty is indicated as the light gray band corresponding to a 95% uncertainty range. the Provisional Maximum Tolerable Daily Intake (PMTDI) is indicated as a red dotted line; according to JECFA fumonisins alone or in combination have a PMTDI of 2 µg/kg bw/day and DON of 1 µg/kg bw/day.

Materials and Methods

Table S4. Summary statistics for the consumption of maize by men and women reported by Wall-Martinez et al., [1].

Group	Food [2]	Average (g maize/person/day)	SD	Distribution	Shape [1]	Scale [1]
Men	Antojitos	46.67	32.81	gamma	2.02	23.06
	Chilaquiles	20.81	15.94	gamma	1.70	12.20
	Tacos	26.62	20.04	gamma	1.76	15.08
	Tortillas	142.9	104.1	gamma	1.88	75.83
Women	Antojitos	30.57	23.57	gamma	1.68	18.17
	Chilaquiles	15.65	10.95	gamma	1.11	7.66
	Tacos	13.67	12.93	gamma	2.04	12.23
	Tortillas	94.2	66.4	gamma	2.01	46.80

[1] Shape and scale calculated from the average and SD reported by Wall-Martinez et al., [1]. [2] Food providing the largest amount of nixtamalized maize per person (g maize/person/day).

Supplementary Data—The computer code repository is available at: https://doi.org/10.5281/zenodo.4031516.

References

1. Wall-Martínez, H., Ramírez-Martínez, A., Wesolek, N., Brabet, C., et al., Statistical analysis of corn consumption for improved mycotoxin exposure estimates for the population of Veracruz City, Mexico. *Food Additives & Contaminants: Part A* **2017**, *34*, 864–879.
